# A Novel Rhodamine B Fluorescent Probe Derived from Carboxymethyl Chitosan for the Selective Detection of Fe^3+^

**DOI:** 10.3390/polym16223206

**Published:** 2024-11-19

**Authors:** Mei Yang, Zixi Tang, Chunwei Yu, Jun Zhang

**Affiliations:** NHC Key Laboratory of Control of Tropical Diseases, School of Tropical Medicine, Hainan Medical University, Haikou 571199, China; yang24364@hainmc.edu.cn (M.Y.); 18358091996@163.com (Z.T.)

**Keywords:** chitosan, rhodamine B, Fe^3+^, fluorescence

## Abstract

In this study, we synthesized a fluorescent material by modifying the C-2 amino group of carboxymethyl chitosan with a rhodamine B derivative, which was proposed and demonstrated using ^1^H NMR and FT-IR measurements. A series of experiments including selectivity, sensitivity, reversibility, pH, and water content were conducted to investigate the fluorometric and colorimetric properties of the grafted polymer. Utilizing a Fe^3+^-induced ring-opening mechanism of the rhodamine B spirolactam, we found that the grafted polymer exhibited a highly selective fluorescence response to Fe^3+^, with enhanced fluorescence at 583 nm compared to other tested metal ions and anions, accompanied by the characteristic absorption peak of rhodamine B that appeared at 561 nm with a noticeable color change from colorless to pink, facilitating visual observation. Additionally, the modified probe, composed of carboxymethyl chitosan, was easily regenerated through treatment with EDTA.

## 1. Introduction

Fe^3+^ plays an essential role in various biological processes, including brain function, gene transcription, immune response, and mammalian reproduction [1]. Deviations in the concentration of Fe^3+^ from its optimal range can lead to serious health issues, including Parkinson’s disease and Alzheimer’s disease [2]. Therefore, the detection of Fe^3+^ is crucial for monitoring and controlling its levels in the biosphere, thereby minimizing potential impacts on human health.

The fluorescence probe method for the selective detection of various biologically and environmentally relevant analytes has been widely studied and applied due to its simplicity, ease of modification, high sensitivity, and non-invasive nature, making it suitable for the real-time analysis of living systems [3,4]. This recognition process typically relies on a straightforward fluorescence enhancement (turn-on) or quenching (turn-off) response. In terms of sensitivity and selectivity, probes that exhibit fluorescence enhancement upon analyte complexation are generally preferred over those that show fluorescence quenching upon analyte binding [5,6]. To date, significant progress has been made regarding the development of various Fe^3+^ fluorescent probes utilizing different fluorophores, including benzimidazole [7], pyrene [8], naphthalimide [9], coumarin [10], and rhodamine [11]. Given the quenching effect induced by the paramagnetism of Fe^3+^ [12], there is considerable interest in designing fluorescent probes that produce highly sensitive “turn-on” fluorescence signals in response to Fe^3+^. Among these fluorophores, rhodamine dyes are particularly prominent in constructing fluorescence-enhanced signal probes. Based on the spirolactam (switch-off) to ring-open amide (switch-on) equilibrium of rhodamine mediated by guest molecules, rhodamine-based probes facilitate a visually observable detection process [13], which is advantageous for practical applications. Numerous rhodamine-based turn-on fluorescent probes for metal ions have been reported [14,15,16]. These fluorescent probes are designed using chemical materials tailored to the characteristics of the analytes. The application of such synthetic probes can be further expanded if they possess attributes such as biocompatibility, biodegradability, and stability across a wide pH range.

Chitosan is a biocompatible and biodegradable biopolymer recognized for its antimicrobial properties and strong adsorption capacity. Its structure features a high number of hydroxyl and amino groups, which provide numerous sites for chemical modifications. This characteristic enables the development of a three-dimensional (3D) fluorescence detection platform for the selective recognition of metal ions [17,18,19]. These unique properties make chitosan highly versatile for applications in medicine, environmental remediation, and food technology.

**Figure 1:**
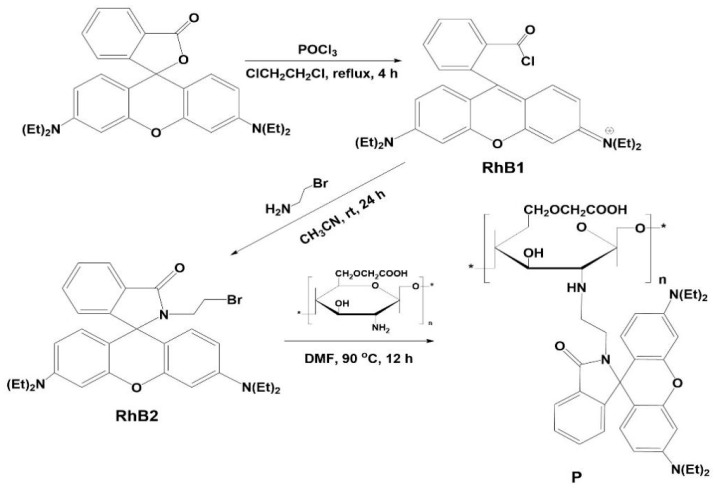
Synthetic route of **P**; “*”: represents the repeated units.

With this in mind, the connection of the chitosan material with a rhodamine-based receptor was synthesized in this paper, as shown in Figure 1. We investigated the properties of the polymer able to act as an off-on fluorescent probe for selective Fe^3+^.

## 2. Materials and Methods

### 2.1. Materials and Instruments

CuCl_2_·2H_2_O, ZnSO_4_, CdCl_2_, CrCl_3_·6H_2_O, FeCl_3_·6H_2_O, NaCl, KCl, CaCl_2_, HgCl_2_, MgCl_2_·6H_2_O, AlCl_3_·6H_2_O, AgNO_3_, NaClO_4_, NaNO_3_, Na_2_CO_3_, KH_2_PO_4_, K_2_HPO_4_, KCl, KI, KBr, anhydrous ethanol, 4-(2-hydroxyethyl)-1-piperazineethanesulfonic acid (HEPES), 1,2-dichloroethane, phosphorus trichloride, acetonitrile, methanol, 2-bromoethylamine hydrochloride, triethylamine, dichloromethane, dimethyl sulfoxide, sodium carbonate, and *N*,*N*-dimethylformamide were all purchased from Tianjin Kemiou Chemical Reagent Co., Ltd. (Tianjin, China). Rhodamine B lactone was purchased from Across International (Beijing, China). Carboxymethyl chitosan, with a degree of carboxylation ≥ 80% and a molecular weight of around 9000, was purchased from Shanghai Yuan Ye Biotechnology Co., Ltd. (Shanghai, China). All reagents used in the experiments were of analytical or chromatographic purity and were not further purified prior to use; water was purified with a Milli-Q system (purified to 18.2 M cm).

Fluorescence emission spectra were obtained using a Hitachi F-4600 spectrofluorometer (Hitachi, Ltd., Chiyoda, Japan), and UV–visible spectra were recorded with a Hitachi U-2910 spectrophotometer (Hitachi, Ltd., Japan). Nuclear magnetic resonance (NMR) spectra were measured with a Bruker AV 400 instrument (Bruker Co., Fällanden, Switzerland), with chemical shifts reported in parts per million (ppm) relative to tetramethylsilane (TMS). Mass spectrometry (MS) was conducted on a Thermo TSQ Quantum Access system coupled with an Agilent 1100 system (Thermo Fisher Scientific Inc., Waltham, MA, USA). Fourier transform infrared spectroscopy (FT-IR) analysis was carried out using a spectrometer (PerkinElmer Inc., Waltham, MA, USA) following the KBr tablet method.

### 2.2. Synthesis of Compounds

Compound RhB1 and RhB2 were synthesized according to reported procedures [20].

Compound N-(rhodamine B) lactam-N’-carboxymethyl chitosan-ethylenediamine (**P**): In a 150 mL round-bottom flask, 0.0529 g of carboxymethyl chitosan (CMCS) was added, followed by 0.3372 g of RhB2 (0.61 mmol), 10 mL of *N*,*N*-dimethylformamide (DMF), and 0.0702 g of anhydrous sodium carbonate (0.66 mmol). The mixture was reacted at 90 °C for 12 h, yielding a transparent light pink solution, which was subsequently cooled to room temperature. After vacuum filtration, the filtrate was evaporated, and ice water was added to precipitate the product. The resulting pink solid was collected and extracted using ethanol as the solvent in a Soxhlet extractor. IR (KBr): 3438.61 cm^−1^, 2920.28 cm^−1^, 1617.18 cm^−1^, 1426.09 cm^−1^, 1310.53 cm^−1^, 1138.12 cm^−1^, 1054.67 cm^−1^, and 725.15 cm^−1^.

### 2.3. Testing Method

The stock solution of probe **P** was prepared at 5000 ppm in DMF. Metal salt and anion solutions (10 mM) were prepared in water. A typical test solution was prepared by placing 50 µL of **P** stock solution (5000 ppm), an appropriate aliquot of each ion solution and 1.5 mL of ethanol into a 5 mL centrifugal tube and diluting the solution to 5 mL with HEPES (20 mM, pH 6.0). The mixtures were equilibrated at room temperature for 30 min before the spectroscopy measurements were recorded. The excitation wavelength of **P** was 530 nm, with both the excitation and emission slits set to 10 nm.

### 2.4. Selectivity Study of **P**

An amount of 50 μL (5000 ppm) of **P** was placed in a test tube, and 5 μL (10 mM) of various common metal ions (Hg^2+^, Ni^2+^, Cu^2+^, Co^2+^, Ca^2+^, Na^+^, K^+^, Cr^3+^, Zn^2+^, Mg^2+^, Al^3+^, Mn^2+^, Pb^2+^, Cd^2+^, Fe^3+^) and anions (NO_3_^−^, SO_4_^2−^, CO_3_^2−^, I^−^, Br^−^, HPO_4_^2−^, H_2_PO_4_^−^, ClO_4_^−^) were added separately. One test tube was used as a blank. The solution was diluted to 5 mL with ethanol–water solution (3:7, *v*:*v*, pH 6.0, 20 mM HEPES) and thoroughly shaken and left to stand in the dark for 30 min before performing fluorescence and UV–vis spectroscopy to examine the selectivity of **P**.

### 2.5. Sensitivity

An amount of 50 µL (5000 ppm) of **P** was added to a 5 mL test tube, followed by the addition of an appropriate volume of Fe^3+^ solution (10 mM). The solution was then diluted to a final volume of 5 mL with ethanol–water solution (3:7, *v*:*v*, pH 6.0, 20 mM HEPES), resulting in Fe^3+^ concentrations ranging from 0 to 100 μM. The mixture was shaken well and allowed to stand for 30 min before conducting fluorescence spectroscopy.

### 2.6. Different Ethanol–Water Ratios

For the investigation of **P**, 50 µL (5000 ppm) of **P** was added to a 5 mL test tube, followed by an equal volume of ethanol. Subsequently, an equal volume of water was added according to the ethanol/water (*v*:*v*) ratios of 0:10, 1:9, 2:8, 3:7, 4:6, 5:5, 6:4, 7:3, 8:2, 9:1, and 10:0. The mixture was then shaken thoroughly and allowed to stand in the dark for 30 min before conducting fluorescence spectroscopy.

For **P**-Fe^3+^ system, 50 µL (5000 ppm) of **P** and 50 µL (10 mM) of Fe^3+^ solution was added to a 5 mL test tube. The same procedure as described for the investigation of **P** was followed.

### 2.7. pH Experiment

For **P**, 50 µL (5000 ppm) of **P** was added to a 5 mL test tube, followed by the addition of 1.5 mL of ethanol. Then, 0.5 mL of 20 mM HEPES buffer solution (pH 4.0, 4.5, 5.0, 5.5, 6.0, 6.5, 7.0, 7.5, 8.0, 8.5, 9.0, 9.5, and 10.0) was added sequentially. After mixing thoroughly, the solution was left to stand in the dark for 30 min before conducting fluorescence spectroscopy.

For **P**-Fe^3+^ system, 50 µL (5000 ppm) of **P** and 50 µL (10 mM) of Fe^3+^ solution were added to a 5 mL test tube. The same procedure as described for the investigation of **P** was followed.

### 2.8. Reversibility

I: 50 µL (5000 ppm) of **P** solution was added to a 5 mL test tube; II: 50 µL (5000 ppm) of **P** solution was added to a 5 mL test tube, followed by the addition of 25 μL (10 mM) of Fe^3+^ solution; III: 50 µL (5000 ppm) of **P** solution was added to a 5 mL test tube, followed by the addition of 25 μL (10 mM) of Fe^3+^ solution and then 100 μL of EDTA (10 mM) solution; IV: 50 µL (5000 ppm) of **P** solution was added to a 5 mL test tube, followed by the addition of 25 μL (10 mM) of Fe^3+^ solution, 100 μL of EDTA (10 mM) solution, and 100 μL of Fe^3+^ solution (10 mM); V: 50 µL (5000 ppm) of **P** solution was added to a 5 mL test tube, followed by the addition of 25 μL (10 mM) of Fe^3+^ solution, 100 μL of EDTA (10 mM) solution, and 200 μL of Fe^3+^ solution (10 mM). These solutions were then diluted to a final volume of 5 mL with an ethanol–water solution (3:7, *v*:*v*, pH 6.0, 20 mM HEPES), respectively. The mixtures were shaken well and left to stand for 30 min before conducting fluorescence spectroscopy.

### 2.9. The Calculation of the Binding Constant

The binding constant for the formation of **P**-Fe^3+^ complex was evaluated using the Benesi–Hildebrand plot [21].

$$\frac{1}{F-F_{0}}=\frac{1}{K(F_{\max}-F_{0}){\left[{\mathrm{Fe}}^{3+}\right]}_{0}^{n}}+\frac{1}{F_{\max}-F_{0}}$$

*F*_0_ represents the fluorescence intensity of **P** in the absence of Fe^3+^, *F* denotes the fluorescence intensity of **P** when Fe^3+^ is present, and *F*_max_ indicates the fluorescence intensity of **P** when an excess of Fe^3+^ is added. The binding constant (*K*) is expressed in units of M^−1^ and is determined from the slope of the linear plot.

## 3. Results and Discussion

### 3.1. Synthesis and Structure Characterization of **P**

**Figure 2:**
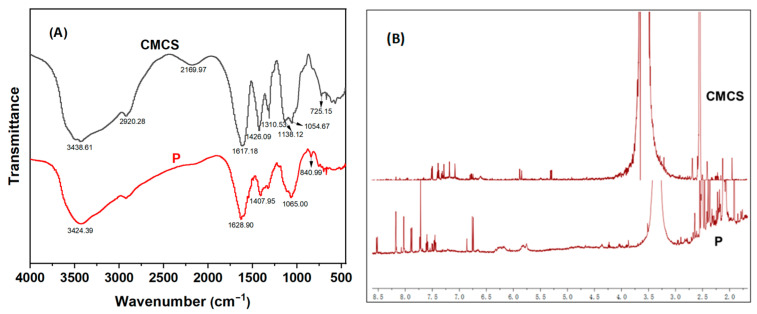
(**A**) FT-IR pattern of CMCS and **P**; (**B**) ^1^H NMR of CMCS and **P**.

After grafting RhB2 onto CMCS through a simple substitution reaction, the structure of **P** was characterized by FT-IR and ^1^H NMR (Figure 2). Compared to the FT-IR spectrum of CMCS (Figure 2A), new absorption peaks appeared at 2920.28 cm^−1^, 1310.53 cm^−1^, 1138.12 cm^−1^, and 725.15 cm^−1^, which were attributed to the stretching vibrations of -C-H and -C=C- in the benzene ring, confirming the successful grafting of RhB2 onto CMCS. To further investigate the effective synthesis of RhB2 with CMCS, ^1^H NMR spectra were also obtained (Figure 2B), showing new peaks around 6.75 to 8.60 ppm attributed to Ar-H, as well as a peak at approximately 1.25 ppm corresponding to -CH_3_, which further supported the formation of **P**.

### 3.2. Application of **P** for the Detection of Fe^3+^

**Figure 3:**
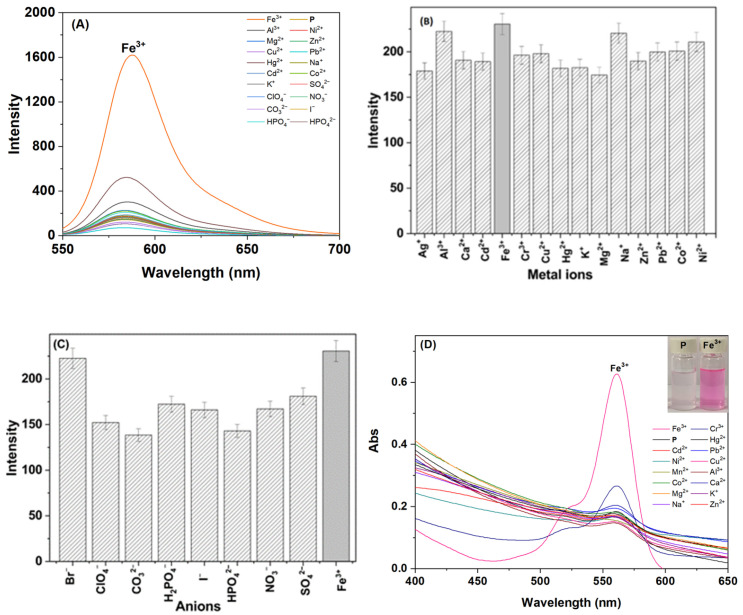
The ability of **P** to recognize Fe^3+^ in an ethanol–water solution (3:7, *v*:*v*, pH 6.0, 20 mM HEPES). (**A**) Fluorescence spectra of **P** (50 ppm) with different ions (100 μM). (**B**) Fluorescence spectra of **P** (50 ppm) to Fe^3+^ (10 μM) and to the mixture of individual other metal ions (10 μM) with Fe^3+^ (10 μM). (**C**) Fluorescence spectra of **P** (50 ppm) to Fe^3+^ (10 μM) and to the mixture of individual other anions (10 μM) with Fe^3+^ (10 μM). (**D**) Absorption spectra of **P** (50 ppm) with different metal ions (100 μM); inset: solution pictures of **P** and **P**-Fe^3+^.

The selectivity of probe **P** for various metal ions and anions in an ethanol–water solution (3:7, *v*:*v*, pH 6.0, 20 mM HEPES) was systematically investigated (Figure 3).

The interactions between probe **P** (50 ppm) and common metal ions and anions (100 μM) were assessed. In this an ethanol–water solution, probe **P** exhibited a cyclic conformation, resulting in relatively weak fluorescence intensity in the range of 550–700 nm. However, upon the addition of Fe^3+^, the cyclic structure of the probe underwent a conformational change to an open-ring state, leading to a significant fluorescence enhancement at 583 nm (Figure 3A). The fluorescence intensity observed in this system was markedly higher than that of other tested metal ions and anions, including Na^+^, K^+^, Ag^+^, Zn^2+^, Cd^2+^, Cu^2+^, Mg^2+^, Ca^2+^, Hg^2+^, Cr^3+^, I^−^, H_2_PO_4_^−^, HPO_4_^2−^, SO_4_^2−^, Br^−^, CO_3_^2−^, NO_3_^−^, and ClO_4_^−^. This indicated that **P** displayed a pronounced selectivity for Fe^3+^ over other ions, demonstrating its potential as a highly efficient and sensitive fluorescent probe for the target metal ion. Moreover, when 1 equiv. of Fe^3+^ was introduced into the solution containing other metal ions and anions (10 μM), as depicted in Figure 3B and Figure 3C, the presence of these other ions did not significantly impact the fluorescence intensity of **P**-Fe^3+^ complex. This further underscored that the synthesized **P** was an excellent and stable fluorescent probe for the reliable detection of Fe^3+^.

In the ethanol–water solution (3:7, *v*:*v*, pH 6.0, 20 mM HEPES), we also investigated the selectivity of probe **P** by examining the effects of various metal ions and anions on its absorption spectra, with the results shown in Figure 3D. Prior to the addition of Fe^3+^, probe **P** (50 ppm) did not display the characteristic absorption peak of rhodamine B in the 400–600 nm range. Upon the addition of Fe^3+^, the characteristic absorption peak of rhodamine B appeared at 561 nm, which was also due to the conversion from the lactam ring (off state) to the open-ring amine (on state). This change was accompanied by a color shift in the solution to pink, enabling “naked eye” detection.

**Figure 4:**
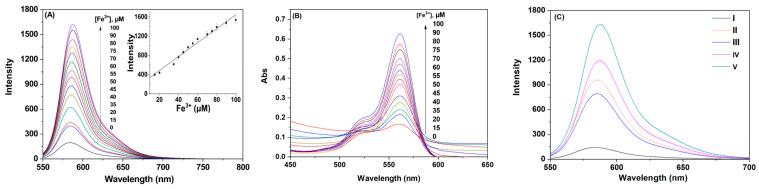
(**A**) Fluorescence spectra of **P** (50 ppm) with various concentrations of Fe^3+^ (0–100 μM) in an ethanol–water solution (3:7, *v*:*v*, pH 6.0, 20 mM HEPES). (**B**) Absorption spectra of **P** (50 ppm) with various concentrations of Fe^3+^ (0–100 μM) in an ethanol–water solution (3:7, *v*:*v*, pH 6.0, 20 mM HEPES). (**C**) Reversibility of **P** in an ethanol–water solution (3:7, *v*:*v*, pH 6.0, 20 mM HEPES): I. **P** (50 ppm); II. **P** (50 ppm) + Fe^3+^ (50 μM); III. **P** (50 ppm) + Fe^3+^ (50 μM) + EDTA (200 μM); IV. **P** (50 ppm) + Fe^3+^ (50 μM) + EDTA (200 μM) + Fe^3+^ (200 μM); V. **P** (50 ppm) + Fe^3+^ (50 μM) + EDTA (200 μM) + Fe^3+^ (400 μM).

To enable **P** to have good practical applications in real environments, we further investigated the effect of different concentrations of Fe^3+^ on the fluorescence spectrum of **P**, as shown in Figure 4. The fluorescence intensity of probe **P** at 583 nm increased systematically with the increasing concentration of Fe^3+^, demonstrating a good linear relationship (Figure 4A). The detection limit of the probe for Fe^3+^ in this medium was 5.0 μM (calculated based on the limit of detection, LOD = 3s_0_/s where s_0_ is the standard deviation of the blank measurement (n = 5) and s is the sensitivity of the calibration curve). Additionally, a good linear relationship is observed within the range of 15–100 μM, indicating that this probe is sensitive enough to detect environmentally relevant levels of Fe^3+^. At the same time, the continuous increase in Fe^3+^ concentration led to a linear enhancement in the intensity of the characteristic absorption peak of rhodamine B at 561 nm, which further demonstrates that probe **P** was an ideal fluorescent probe for Fe^3+^ (Figure 4B).

Additionally, we investigated the effect of EDTA on the fluorescence spectrum of the **P**-Fe^3+^ system (Figure 4C). The results revealed that in the ethanol–water solution (3:7, *v*:*v*, pH 6.0, 20 mM HEPES), the fluorescence intensity of probe **P** was relatively weak, attributed to its cyclic conformation, illustrated in line I. Upon the addition of Fe^3+^, probe **P** selectively recognized Fe^3+^ and transitioned to an open-ring conformation, resulting in a significant enhancement of fluorescence intensity at 585 nm in the **P**-Fe^3+^ system, reflected in line II. Following the addition of EDTA, a decrease in fluorescence intensity was observed, indicating that only a small fraction of Fe^3+^ was complexed by the EDTA reagent, which led to the reduction in fluorescence intensity, as demonstrated in line III. When an excess of Fe^3+^ was introduced, the fluorescence intensity of the probe at 585 nm not only recovered but also exhibited a slight increase, as shown in line IV. Further additions of excess Fe^3+^ continued to enhance the fluorescence intensity (line V). This behavior demonstrated that the binding of Fe^3+^ to the probe was a reversible process, facilitating the cyclic use of the probe. Thus, we concluded that the enhancement of the fluorescence signal resulted from the coordination of the ions with the probe [22,23], rather than from catalytic action by the ions.

Table 1 summarized the properties and applications of typical Fe^3+^ fluorescent materials. Various probes grafted with chitosan, lignin, BSA, ethylcellulose, cellulose, and other polymers exhibit different properties for Fe^3+^ detection, including good selectivity and high sensitivity [24,25,26,27], as well as promising potential applications [24,26,28,29]. However, most Fe^3+^ probes were of the fluorescence quenching type [24,25,26,27,28,29,30,31], which often suffer from limitations such as low sensitivity [30,31] and a narrow linear range [24,26]. In contrast, the chitosan-based functional fluorescent material **P** belonged to the fluorescence-enhanced category, demonstrating excellent selectivity, high sensitivity, and a wide linear range.

### 3.3. Experimental Condition Optimization

**Figure 5:**
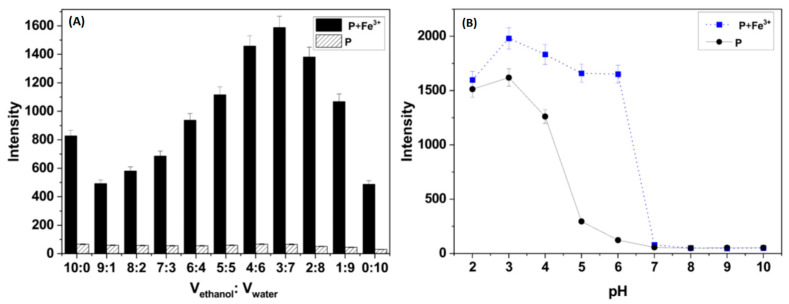
(**A**) Influence of water volume on the fluorescence spectra of **P** (50 ppm) and **P** (50 ppm) plus Fe^3+^ (100 μM). (**B**) Influences of pH on the fluorescence spectra of **P** (50 ppm) and **P** (50 ppm) plus Fe^3+^ (100 μM) in ethanol–water solution (3:7, *v*:*v*).

The effect of water content on fluorescence was investigated, as shown in Figure 5A. As the volume fraction of water increased, the fluorescence emission of the **P**-Fe^3+^ system was significantly influenced, initially exhibiting an enhancement followed by a decrease. Notably, even in pure water, the probe still responded to Fe^3+^, indicating that the incorporation of carboxymethyl chitosan into the rhodamine derivative RhB2 significantly improved its water solubility. The fluorescence intensity ratio of the **P**-Fe^3+^ system to that of probe **P** was maximized at a 3:7 (*v*:*v*) ethanol–water mixture, leading to the decision to use this ethanol–water ratio for subsequent experiments. Additionally, the effect of pH on the system was explored to evaluate the sensing capability for Fe^3+^, as depicted in Figure 5B. When the pH was below 6.0, the fluorescence intensity of both **P** and the **P**-Fe^3+^ complex at 585 nm gradually increased, possibly due to the protonation of the probe. However, a decrease in fluorescence intensity was observed when **P** was mixed with Fe^3+^ in the pH range of 6.0 to 10.0, likely because Fe^3+^ formed iron hydroxide precipitated under alkaline conditions. These pH-controlled measurements indicated that **P** was effective in weakly acidic to neutral environments, which was advantageous for practical applications. To further explore the interaction between **P** and Fe^3+^, all measurements were conducted in an ethanol–water solution (3:7, *v*:*v*, pH 6.0, 20 mM HEPES).

### 3.4. Reaction Mechanism Research

**Figure 6:**
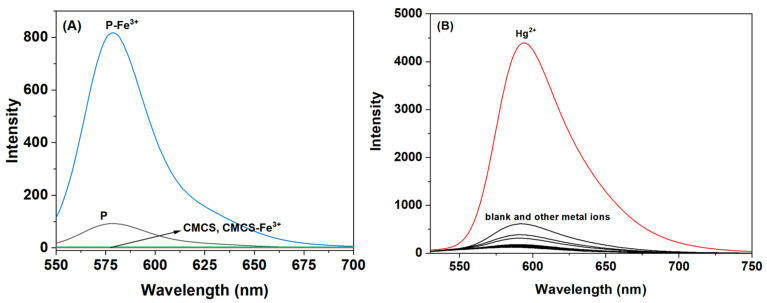
(**A**) Comparison of selectivity of **P** and CMCS (50 ppm) for Fe^3+^ (100 µM) in ethanol. (**B**) Fluorescence spectra of RhB2 (50 ppm) with different metal ions (100 μM) in ethanol.

**Figure 7:**
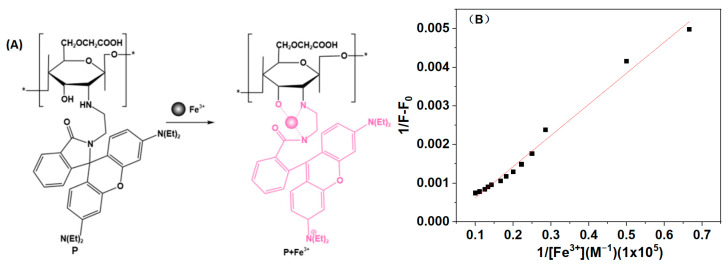
Proposed binding mode of **P** and Fe^3+^; “*”: represents the repeated units. (**A**) The recognition mechanism of **P** with Fe^3+^. (**B**) Benesi-Hildebrand plot of **P**, assuming 1:1 stoichiometry for association between **P** and Fe^3+^.

To assess the specific recognition of the self-synthesized probe **P** for Fe^3+^, we examined the interactions between probe **P**, carboxymethyl chitosan, and iron ions, respectively, as illustrated in Figure 6A. The results reveal that a significant fluorescence intensity at 585 nm was observed exclusively in the **P**-Fe^3+^ system. Additionally, we evaluated the selectivity of the rhodamine derivative RhB2 for common metal ions in ethanol, as shown in Figure 6B. RhB2 demonstrated a good selectivity for Hg^2+^ but exhibited relatively weak fluorescence intensity for Fe^3+^ at 585 nm. This indicated that only our synthesized probe **P** can effectively complex with Fe^3+^, transitioning from a cyclic to an open-ring conformation, which resulted in strong fluorescence at 585 nm. This finding suggested that carboxymethyl chitosan provided essential recognition sites. Furthermore, we investigated the effect of water content on the fluorescence signal of **P** in recognizing Fe^3+^. We found that even in pure water, probe **P** still displayed a significant fluorescence signal for Fe^3+^, whereas RhB2 showed no recognition capability for Hg^2+^ in pure water. It demonstrated that the incorporation of carboxymethyl chitosan onto RhB2 enhanced the water solubility of the probe. Based on the above-mentioned results, the recognition mechanism was proposed as shown in Figure 7A. -OH and -NH- from chitosan and -NH- from RhB2 participated in the coordination process, which caused the opening of the spiro ring of RhB2 part. This mode was also supported by Benesi–Hildebrand plot method (Figure 7B), the binding constant of **P** with Fe^3+^ was determined to be 9.2 × 10^3^ M^−1^ based on a 1:1 complex [21].

## 4. Conclusions

In conclusion, we synthesized a novel rhodamine-based “off–on” fluorescent probe featuring a carboxymethyl chitosan moiety for the selective and sensitive detection of Fe^3+^. The probe demonstrated a significant enhancement in fluorescence intensity, along with a distinct color change from colorless to pink upon binding with Fe^3+^, which was not influenced by the presence of other common competing metal ions. The incorporation of carboxymethyl chitosan notably improved the probe’s water solubility and selectivity. We believe that this design concept will serve as a valuable reference for the development of new chitosan-based probes targeting transition metal ions.

## Figures and Tables

**Table 1:** Performances comparison of various grafted fluorescent probes for Fe^3+^.

Fluorescent Probes	Off/On	Linear Range (µM)	LOD (µM)	Testing Media	Applications	Refs.
Chitosan-based naphthalimide derivative	Off	NA	0.211	H_2_O-DMSO (1:1, *v*:*v*, pH 7.4)	spinach, blood, ice, oysters, water	[24]
Chitosan-based BODIPY derivative	Off	0–120/0–80	1.79/1.25	H_2_O-DMF (4:1, *v*:*v*, pH 7.0)	water	[28]
Chitosan-based rhodamine B derivative	Off	20–80	5	H_2_O	NA	[30]
Chitosan-based urolithin B derivative	Off	80–100	NA	H_2_O-CH_3_COOH (99:1, *v*:*v*)	NA	[31]
Lignin-based BODIPY derivative	Off	0–25	0.21/0.11	H_2_O/H_2_O-DMSO (2:1, *v*:*v*)	NA	[25]
BSA-CuNCs	Off	0.2–2.4	0.01	H_2_O	water, blood	[26]
Ethylcellulose-based flavonol derivative	Off	0–20	0.265	H_2_O-DMSO (9:1, *v*:*v*)	water	[29]
Cellulose-based naphthalimide derivative	Off	2–35	0.90	H_2_O-DMSO (5:1, *v*:*v*)	NA	[27]
Chitosan-based rhodamine derivative	On	15–100	5	H_2_O-ethanol (7:3, *v*:*v*, pH 6.0)	NA	This work

## Data Availability

The original contributions presented in the study are included in the article, further inquiries can be directed to the corresponding author.
